# Rectal fistula induced artificial vascular graft infection: A case report

**DOI:** 10.1097/MD.0000000000041188

**Published:** 2025-01-03

**Authors:** Honghua Zhou, Kanghui Dai, Zhinan Ju, Liqun Wan, Guangmao Zhou, Jiehua Qiu

**Affiliations:** a Department of General Surgery, Jiujiang University Affiliated Hospital, Jiujiang, Jiangxi, China; b Department of Vascular Surgery, The Second Affiliated Hospital of Nanchang University, Nanchang, Jiangxi, China.

**Keywords:** rectal fistula, risk factors, vascular graft infection, vascular surgeon

## Abstract

**Rationale:**

Artificial vascular graft infection (AVGI) is a rare but severe complication in vascular surgery, often associated with high morbidity and mortality. This case report highlights a unique instance of AVGI caused by a rectal fistula, emphasizing the diagnostic challenges and management strategies in such a rare scenario.

**Patient concerns:**

A 60-year-old female presented with low-grade fever and purulent discharge from an abdominal incision 1 year after antibiotic therapy had failed to resolve the infection. She had a history of aortofemoral dacron graft bypass 6 years prior, complicated by a rectal hematoma due to trauma.

**Diagnoses:**

The patient was diagnosed with a rectal fistula, AVGI, and perivascular sinus formation. Diagnostic workup revealed the presence of Escherichia coli infection.

**Interventions:**

The patient underwent graft excision and debridement. No vascular reconstruction was necessary due to the presence of sufficient collateral circulation. This intervention was performed following the failure of conservative antibiotic therapy.

**Outcomes:**

Postoperative recovery was uneventful, with normalization of inflammatory markers and no signs of limb ischemia upon follow-up. The patient’s symptoms resolved without complications.

**Lessons:**

This case underscores the importance of early diagnosis using imaging, particularly in patients with vascular prosthetic grafts who may have adjacent organ injuries such as a rectal fistula. It also highlights the need for tailored surgical approaches and the significance of regular follow-up in managing AVGI.

## 1. Introduction

As the surgical techniques and interventional radiology advances, the number of patients with artificial vascular graft is on the rise. Complications of the use of artificial vascular grafts are also increasing reported. And one of the most dangerous and complex complications is artificial vascular graft infection (AVGI). It is rare, but can be a severe and life-threatening complication in vascular surgery. The catastrophic results include death and limb loss. Here, we report 1 infrequent case of AVGI caused by a rectal fistula.

## 2. Case report

**Figure 1.**
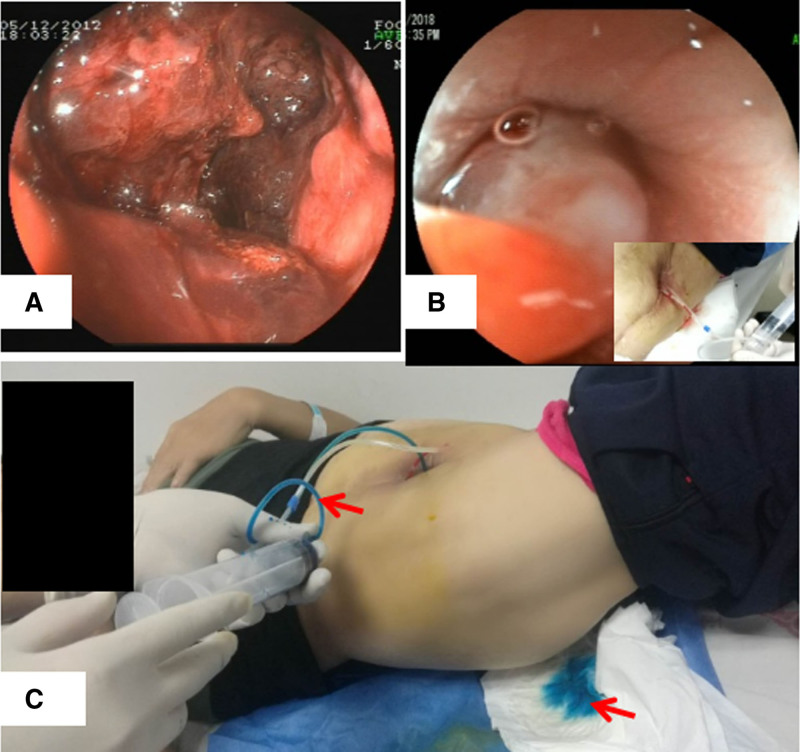
The colonoscopy and the physical examination. (A) The colonoscopy after the accident showed a rectal hematoma. The rectal hematoma detected 6 months after the first operation (artificial blood vessel transplantation). (B) The colonoscopy before the graft removal showed a rectal fistula up to about ten cm from the anus. Air was insufflated through the catheter on the abdominal cavity. (C) Injection of methylene blue solution through a catheter on the sinus and methylene blue flows out from the anus.

A 60-year old female was admitted to our hospital with a low-grade fever and purulent secretion from the incision at the right lower abdomen. She had suffered from the symptoms for about 1 year and underwent an antibiotic therapy in the local hospital but showed little effect. Six years ago, the patient came to our hospital with the right low extremity intermittent claudication and rest pain and then received a conventional aortofemoral dacron graft bypass because the pre-operation computed tomographic angiography (CTA) showed obstruction of the right external iliac artery. She recovered well after the surgery and was discharged without claudication. It is noteworthy that 6 months after the operation, she was involved in an accident which triggered a rectal hematoma (Fig. 1A) and recovered under conservative treatment.

**Figure 2.**
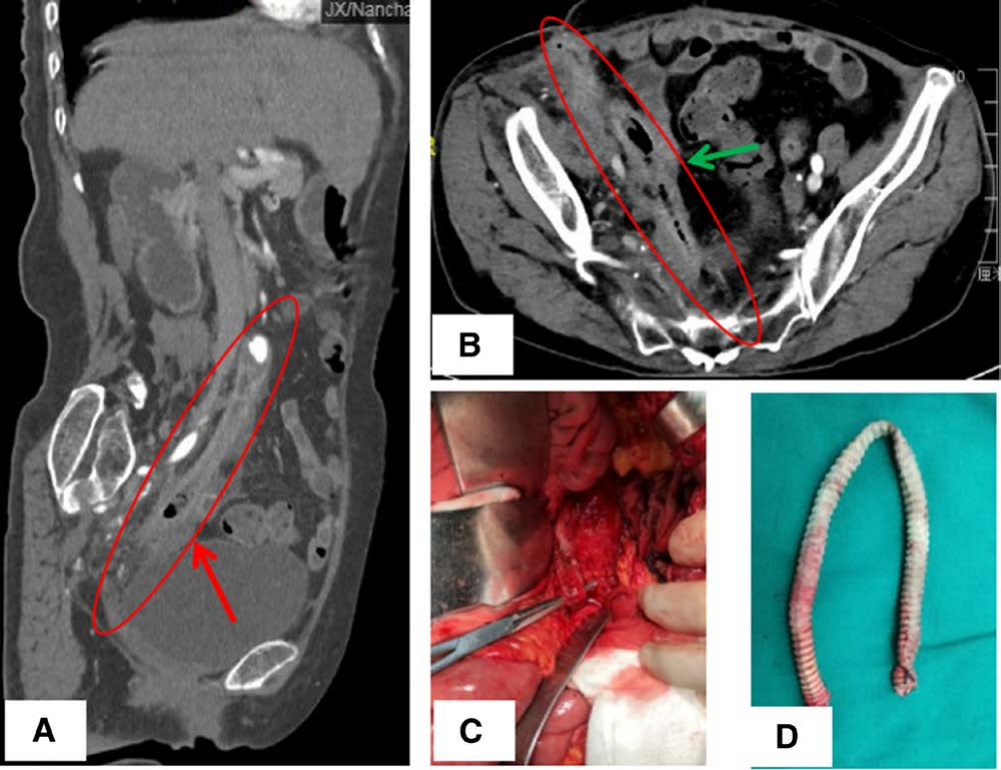
The CT examination and the integrity of infected artificial graft. (A, B) Before the second surgery (removal of vascular grafts), computed tomographic angiography (CTA) showed ectopic gas around the artificial blood vessels, and the right lower anterior abdominal sinus was formed. (C) Infected vascular grafts were found in the second operation. (D) Excised infected vascular graft at the second surgery. CTA = computed tomographic angiography.

The laboratory tests showed elevated peripheral white blood cell count (9.74 × 10^9^/L), neutrophil (7.2 × 10^9^/L), C-reactive protein (over 200 mg/L), erythrocyte sedimentation rate (24 mm/hour) and procalcitonin (2.71 ng/mL). The purulent secretion was positive for Escherichia coli. Colonoscopy revealed a rectal fistula the same place where the rectal hematoma was spotted 5 years ago due to the accident, and an air test and a methylene blue test confirmed a sinus between the rectum and the abdominal wall (Fig. 1B, C). A CTA examination showed the right iliac prosthesis was occlusive with perivascular infection and a sinus formation in right anterior lower abdomen (Fig. 2A, B). Therefore, we suspect a rectal fistula induced AVGI. After peri-operative preparation, the patient was treated with graft excision and debridement, but without artery reconstruction. Intraoperative, a peri-graft intestinal adhesion was evident and segments of intestinal wall participate in a composition of the sinus formation. The infected vascular graft was found and removed (Fig. 2C, D). About 2 months after the operation, the patient’s value of C-reactive protein, erythrocyte sedimentation rate and procalcitonin dropped to normal ranges. She recovered well with no signs of right extremity lower ischemia and continued to receive postoperative anti-infective treatment.

## 3. Discussion

AVGI remains a serious and challenging complication in vascular surgery, with incidences ranging from 1% to 6%.^[1]^ We report an elderly female who developed a vascular graft infection due to a rectal fistula 6 years after the implantation of vascular graft. To the best of our knowledge, the present case is the first description of a rectal fistula induced AVGI.

The causes of AVGI are considered multifactorial, including contamination and direct seeding during implantation, extension from a wound infection adjacent to the graft, or hematogenous seeding. Staphylococcus aureus and Staphylococcus epidermidis are the most common pathogenic bacteria.^[2]^ In our case, a culture of the purulent secretion detected Escherichia coli, a Gram-negative enteric organism abundant in intestinal tracts, which is not common in AVGIs but provides as a certification for our diagnosis.

AVGIs are not easy to diagnose since the clinical presentations are often highly variable and nonspecific manifestations such as fever, back pain and elevated bio-inflammatory markers. Therefore, the use of imaging studies is essential. Imaging modalities including ultrasound, computed tomographic angiography (CTA) and 18F-fluorodeoxyglucose positron emission tomography/computed tomography are most commonly used.^[3]^ In recent years, a series of studies indicates that 18F-fluorodeoxyglucose positron emission tomography/computed tomography is a promising tool contributing to early diagnosis of vascular graft infection. It shows the intensity and patterns of FDG uptake which closely related to the evaluation of vascular graft infection.^[4]^ In general, CT is considered the diagnostic imaging test for AVGI and carries a sensitivity of 94% and a specificity of 85%.^[5]^ In this case, CTA revealed the presence of ectopic gas and peri-graft soft tissue enhancement. Other criteria used for AVGI diagnosis include peri-graft fluid, soft-tissue attenuation or pseudoaneurysm.^[5]^

For patients with AVGIs, early removal of infected grafts with or without revascularization is the preferred treatment. According to literature reports, AVGI requires multidisciplinary management, and treatment usually involves surgery and systemic antibiotics.^[6]^ Surgical treatment includes complete removal of the infected graft and surrounding tissue of the prosthesis, as well as revascularization by in situ or anatomical bypass.^[7]^ Our patients immediately underwent vascular graft resection and debridement once the diagnosis was identified. Since the patient’s preoperative CTA showed the collateral circulation of the lower limbs was abundant and there were no evident signs of ischemia in lower extremity, we did not perform revascularization in order to prevent secondary infections. We recommend that whether to perform reconstruction should depend on the presence of limb ischemia, and reconstruction may not be compulsory in patients with sufficient collateral circulation.

## 4. Result

The patient, a 60-year-old female with a history of aortofemoral dacron graft bypass, presented with persistent low-grade fever and purulent discharge, unresponsive to 1 year of antibiotic treatment. Laboratory tests revealed elevated inflammatory markers, and cultures identified Escherichia coli as the causative pathogen. Imaging studies, including colonoscopy and computed tomographic angiography (CTA), confirmed a rectal fistula and perivascular sinus formation, leading to the diagnosis of rectal fistula-induced artificial vascular graft infection (AVGI). The patient underwent graft excision and debridement without vascular reconstruction due to adequate collateral circulation. Postoperatively, inflammatory markers normalized, and no ischemic symptoms were observed at follow-up. These results highlight the feasibility of managing AVGI without revascularization in the presence of sufficient collateral circulation, emphasizing the importance of early diagnosis and individualized treatment strategies.

## 5. Limitation

This study has several limitations. As a single case report, its findings are not generalizable, and larger cohort studies are needed to validate the conclusions.

## 6. Conclusion

A high degree of alert is necessary when patients with vascular prosthetic grafts encountered adjacent organs injuries after graft implantation surgery. In this case, the severe implication of graft infection may be prevented with the rectal hematoma thoroughly evacuated. Therefore, we strongly emphasize the need to risk factor prevention and regular follow-up in these patients.

## Acknowledgments

We express our gratitude to all the medical personnel in vascular surgery, including the invaluable contributions of nurses and doctors. All authors declare that they have no conflict of interest.

## Author contributions

**Data curation:** Honghua Zhou, Kanghui Dai, Zhinan Ju.

**Methodology:** Jiehua Qiu.

**Project administration:** Jiehua Qiu.

**Writing – original draft:** Honghua Zhou, Kanghui Dai.

**Writing – review & editing:** Liqun Wan, Guangmao Zhou, Jiehua Qiu.
